# Speculative Social Science Fiction of Digitalization in Higher Education: From What Is to What Could Be

**DOI:** 10.1007/s42438-021-00260-6

**Published:** 2021-10-25

**Authors:** Juha Suoranta, Marko Teräs, Hanna Teräs, Petar Jandrić, Susan Ledger, Felicitas Macgilchrist, Paul Prinsloo

**Affiliations:** 1 Tampere University, Tampere, Finland; 2 Zagreb University of Applied Sciences, Zagreb, Croatia; 3 University of Wolverhampton, Wolverhampton, UK; 4 The University of Newcastle, Callaghan, Australia; 5 Georg Eckert Institute of International Textbook Studies, Braunschweig, Germany; 6 Institute for Educational Science, University of Goettingen, Goettingen, Germany; 7 University of South Africa, Pretoria, South Africa

**Keywords:** Digitalization, Datafication, Educational technology, Higher education, Imagination, Karl Marx, Qualitative methodology, Postdigital

## Introduction: Unleashing Teachers’ Imagination (Juha Suoranta, Marko Teräs, Hanna Teräs)

Digitalization and datafication are reshaping roles and practices in higher education. The Covid-19 pandemic has accelerated this process through the massive increase in the use of educational technology (EdTech) (Teräs et al. 2020). As a part of this development, higher education is becoming increasingly data driven. Simultaneously, attempts to predict and prepare for future scenarios in education are driven by intergovernmental organization reports from OECD and UNESCO, in addition to national initiatives in digital education (Suoranta et al. 2022). In these future scenarios, datafication, big data, learning analytics, and artificial intelligence promise more efficient and predictable higher education.

However, critical voices in educational and social sciences have warned that datafication narrows the discourse and the scope of complex concepts such as teaching and learning (Jarke and Breiter 2019; Knox et al. 2020; Manolev et al. 2019; Mirrlees and Alvi 2020). Moreover, the visions and practices of data-driven education tend to reduce teachers and the students in higher education to mere objects of digitalization, rather than seeing them as active subjects participating in the shaping of digital futures (e.g., Saari and Säntti 2018; Broughan and Prinsloo 2019).

It was Marx who stated already in 1858 that

once adopted into the production process of capital, the means of labour passes through different metamorphoses, whose culmination is the machine, or rather, an automatic system of machinery (system of machinery: the automatic one is merely its most complete, most adequate form, and alone transforms machinery into a system), set in motion by an automaton, a moving power that moves itself; this automaton consisting of numerous mechanical and intellectual organs, so that the workers themselves are cast merely as its conscious linkages. (Marx 1973: 614)

Higher education teachers are becoming perfect examples of Marx’s description of the workers cast merely as conscious linkages of the automaton. Currently, we live in an era where questions, such as shall or should ‘the automatic system of machinery’ (in our case, digitalization) replace the teacher, have become relevant (see Selwyn et al. 2020).

These questions have materialized in the digitalization of higher education, as the case of Professor François-Marc Gagnon strikingly demonstrates: Gagnon taught in an online course while already dead (Fleming 2021; Matthews 2021). To Peter Fleming (2021), the incident marked ‘the dirty flipside of the edtech trend that’s transforming higher education, concealed behind a new wave of corporate buzzwords: blended learning, hybrid instruction; digital scaffolding; synchronous and asynchronous learning; micro-credentials and so-on’.

The current transformation of contemporary higher education is characterized by managerialism (Poutanen et al. 2020), EdTech market lobbying (Mirrlees and Alvi 2020; Williamson 2021), and technological determinism (Hayes and Jandrić 2014). Technological determinism appears in the discourse of digitalization and datafication in disguise of positive potential to fix the supposedly ‘broken’ education. EdTech corporations, supranational educational lobbying organizations (OECD perhaps as the most influential), state bureaucratic apparatuses, and managerial administrators in the universities create deterministic policies on digitalization, usually without engaging higher education teachers in the open development of educational futures.

These actors often outline and predict a utopian technological future (for economic gains) without listening to educators’ points of view. Therefore, there is a clear need for new educational and political imaginaries and inquiries in which higher education teachers can actively participate in imagining digital futures of higher education. Thus, we argue that teachers as experts in teaching and learning should be more heavily involved in evaluating and shaping the digitalization of higher education.

(Speculative) social science fiction is not a new approach in social sciences (Gerlach and Hamilton, 2003; Lackey 1994). The power of fiction as a method has also been noticed in education (Clough 2002; Facer 2011). Furthermore, and perhaps in the crossroads of social sciences and education, it has recently emerged as a promising approach to engage teachers and students in imagining different futures of digitalization in education (for example, Macgilchrist et al. 2020; Selwyn et al. 2020; see also *Learning, Media and Technology* journal special issue on speculative futures^1^).

The concept of imagination is methodologically fundamental in the social sciences we seek to advance. Imagination is ‘an ongoing relationship and material capacity constituted by social interactions between people’ (Shukaitis 2009: 10). Its aim ‘is not to keep a mirror in front of people’s faces and show them how they look, but to explore ideas and thinking’ (Eskola 1984: 29). Thus, it provides views of what we can be and can become.

Many authors have emphasized originally Marx’s (1887: 127) idea of the power of imagination (‘what distinguishes the worst architect from the best of bees is this, that the architect raises his structure in imagination before he erects it in reality’) in keeping hope alive and striving for social transformation (e.g., Appadurai 2000; Bloch 1995; Castoriadis 1987; Eskola 1988; Freire 2018; Fromm 1968; Graeber 2007; Jandrić and McLaren 2020; Mills 2000; Rorty 1999; Stetsenko 2020). Whereas C. Wright Mills (2000) believed that sociological imagination is the most needed quality of mind for social scientists, Richard Rorty (1999: 87) emphasized that imagination is ‘the source both of new scientific pictures of the physical universe and new conceptions of possible communities’.

We wholeheartedly agree with Stetsenko (2020: 728), who stresses that an essential part of inquiry ‘includes elaborating our sought-after futures and endpoints through debates and deliberations grounded in not only critique, but also in political imagination that is part and parcel of the hard work of transforming the status quo in realizing the future, and thus, ourselves and the world’. Thus, in studying digitalization in higher education, we need to release teachers’ imaginations and invent new social imaginaries of digitalization in higher education with them. We need to switch from ‘what is’ to ‘what is not yet’, but what could be. In this way, imagination allows teachers ‘to see and perform testing actions on the untested feasibility’ and activate their creative power (Dubin and Prins 2011: 37; Freire 2005).

In unleashing teachers’ imagination, we invite them to participate in future workshops to consider possible future scenarios concerning their work; that is, ‘to construct, in the imagination, a model or blueprint of the task to be performed, prior to its performance’ (Ingold 1983: 2).^2^ We also ask them to write short stories and imagine digitalized futures in higher education. Future workshops (Jungk and Müllert 1987) and the method of empathy-based stories (see Wallin et al. 2019; Särkelä and Suoranta 2020) are among the methods we believe can bring into fore unexpected insights that spark imagination (Wallin et al. 2019). These empirical methods can open eyes to ‘the real possibilities we have in this world, but which can only be foreseen by the power of imagination and be carried into effect through action’ (Eskola 1988: 256–261).

## To Run in The Right Direction (Petar Jandrić)

Knowledge development is always situational. After she married the wealthy merchant William and was expected to hold frequent dinner parties, Josephine Cochran wowed: ‘If nobody else is going to invent a [mechanical] dishwashing machine, I’ll do it myself.’ (in Goodrich 2020) The Second World War and later the Cold War were the main reasons for abundant funding of nuclear research, resulting in many technologies which are now in everyday use. Today, the Covid-19 pandemic is responsible for significant growth in many research fields from medicine to law. Directly affecting billions of learners, and indirectly affecting many more people in their communities, the switch to ‘emergency remote learning’ (Hodges et al. 2020) has been one of the most ubiquitous disturbances in our pre-pandemic ways of life. It is therefore hardly a surprise that education researchers are now at the forefront of developing theories and practices suitable for a ‘new normal’.

These research efforts take up various forms and shapes. Within our neoliberal system of research support and funding, however, this abundance of new ideas gets increasingly channelled into few common research strands. One popular strand is the expert advice aimed at helping teachers and learners to cope with pandemic conditions (see Rapanta et al. 2020, 2021). Another popular strand is an inquiry into ways in which teachers and learners have coped with pandemic lockdowns (Jandrić et al. 2021). And yet another popular strand is the development of new EdTech solutions, resulting in the development of new sellers’ markets (Teräs et al. 2020). Conducted with similar vigour within the academia and in EdTech corporate research centres, all these strands share a common focus on educational futures. In our uncertain and precarious pandemic moment, such research focus is hardly a surprise. Yet the central question is, how are we going to shape and use these insights?

In our post-truth world, the question of proper usage of our research insights is far from trivial. A typical case in point is the anti-vaccination movement, which often supports its ridiculous conclusions by perfectly sound research (MacKenzie et al. 2021). However, the question of shaping research gets often neglected, and yet it is important because educational research is never neutral. For instance, when Peter Thiel speaks of looking at ‘what’s going on fundamentally with institutions, with our society and then look for ways to make them better, he refers to education as a billion-dollar industry, and his ideas about improving education are primarily driven by his own profits (Davis et al. 2011). When critical pedagogues talk about improving education, their ideas can almost always be summed up in demands for more public expenditure. To avoid such self-fulfilling prophecies, shows David Kupferman (2021), ‘[w]e need to dream up what comes next and let our imaginations run wild, so that we begin to anticipate the various potentials of postdigital educational futures, rather than backing into them and hoping that we get it right’.

Running wild is important yet far from enough. We can run wild in different directions, and our initial choice of direction will strongly influence the results of our research. With this in mind, I believe that our research in speculative social science fiction of digitalization in higher education should be unapologetically built on a normative framework focused on emancipation, social justice, and other important messages championed by the critical pedagogy movement. It is only through a combination of proper research shaping and proper research usage, that our speculative social science fiction can contribute to the development of a ‘new normal’ in which education will fulfil its mission to make the world a better place.

## And to Fail as We Run (Felicitas Macgilchrist)

How does *failure* figure in imaginations of digital futures, as higher education is trying to make the world a better place? The affirmative enthusiasm of the EdTech industry orients primarily to ‘success’: digital technology will—in this view—help students to reach their potential, enable instructors to support, motivate and teach successfully, and facilitate institutions to lead their faculty, staff, and students into a successful future. Similarly, models and frameworks for twenty-first-century skills, four-dimensional education, or future skills aim to help us to become thriving, fulfilled, successful humans. The future in these scenarios looks bright; these tools and practices will help the future generation of students to be successful adults.

But what counts as ‘success,’ and for whom? Whose priorities are being imagined? A social science fiction methodology, as outlined above, with future workshops and empathy-based storytelling, invites higher education teachers to imagine alternative futures for digital higher education. But will these also be oriented to success? Or might they, in line with postdigital thinking, drag ‘digitalisation and the digital—kicking and screaming—down from its discursive celestial, ethereal home and into the mud’ (Ryberg in Jandrić et al. 2019: 166)? Will they include versions of what Jack Halberstam (2011) has called the ‘queer art of failure’?

In one sense, this refers to the ‘failure to be normal’ (Muñoz 2019: 172), i.e., to live and think and teach and learn without dominant norms and normativity. Referring to speculative utopian thinking, Muñoz (2019: 173) emphasizes that utopia ‘can never be prescriptive and is always destined to fail’. Precisely therein lies its potential. Failure is also about failing to participate in systems of values that prioritize achievement, success, and thereby conformity. This includes the failure to domesticate difference into normed and smoothed forms of inclusive education (Mitchell et al. 2014). The utopian potential of failure lies in its potential as ‘a mode of resistance, intervention, speculation and queer world making’ (Takemoto 2016: 86).

This is, however, no simplistic celebration of failure. As scholars and practitioners attend to failure as a mode of imagining other ways of being, we need to ‘acknowledge the *pleasures* of failure—embodied in choices to stand apart from social norms of gender, sexuality, reprocentricity, and romantic affiliation—and the *distress* of failures embodied in lives gone haywire, symptoms run rampant, personal lives devolving into uninhabitable havoc’ (Johnson 2015: 264). Failure as the ‘refusal of legibility’ within majority normativity is also a ‘bleak territory’ of loss, of ‘unbeing and unbecoming’ (Halberstam 2011: 23, 88).

This kind of failure, it should be clear, is not about ‘learning from mistakes’. An oft-voiced imperative in educational and EdTech spaces, learning from mistakes also orients to success. We experiment, test, try out, stumble, make mistakes, fall. Then — in this metaphor — we pick ourselves up, dust ourselves off, reflect on our mistakes, and move onwards to success. The queer art of failure is instead about the struggle for *other* futures; about critically assessing the politics and positionality of achievement; about exploring how failure is both bleak and hopeful.

In all, I wonder if (and if so, how) a social science fiction methodology can interrupt ‘success’ as a guiding frame for many higher education scholars, practitioners, and activists in the twenty-first century.

## Reflect on Past Practice to Imagine Creative Futures (Susan Ledger)

Educators’ creativity and imaginaries are often constrained by policies, assessment schedules, and context-based forced practices. Daily decisions on teaching and learning are often limited by the knowledge of, and confidence with new technologies, a willingness to be creative, or the ability to apply learning to real-life contexts. However, educators remain the experts in teaching and learning and should be more involved in evaluating and shaping the digitalization of schooling and higher education. Rather than being driven by EdTech profit-making businesses and emerging technological tools, educators should assert themselves by exploring the impact of EdTech on the teaching and learning process (Ledger 2020; Mishra and Koehler 2006).

Those responsible for shaping teaching and learning are at the ‘chalkface’ of technological adoption and change and should not adopt technology simply because it is available. Technology needs to be suitable. Pedagogical scrutiny is required within this ever-changing learning environment. The affordances of new and emerging technologies require critique by teachers’. Critical reflection and collaborative efforts are necessary to assess the overall purpose and usability of ever-evolving digital technologies. Southgate (2020: 7) call for ongoing scrutiny to ensure that ‘technology is used for the benefit of students, educators, communities, and society more broadly’.

Teachers’ professional identities and teaching practices draw on reflective pedagogies (Dewey 1933; Schön 1983; Zeichner 1994; Sellars 2012), diagnostic approaches (Reynolds and Fletcher-Janzen 2007), and cultures of learning through 'sayings, doings and relatings’ (Kemmis et al. 2014). These foundational beliefs have been challenged by international organizations such as OECD. Among its many initiatives on digitalization, project Learning Compass 2030^3^ demands a new type of educator (and worker), ‘one that is self-initiated and collaborative; responsive and reactive’ and ‘able to interface with technology and communicate and is capable of creating new social identities’ (Bahr and Mellor 2016: iv).

'The best way to predict the future is to create it.' ~ Peter Drucker. Creative options in education require educators to be activists and active change agents. We can draw from the past and use new technologies to design learning activities that prepare students for digital futures. An excellent example of this is combining traditional micro-teaching practices (Allen and Eve 1968) with mixed reality learning environments reimagined as micro-teaching 2.0 (Ledger and Fischetti 2020). This initiative uses technology as a tool to link theory to practice.  Technology offers learning environments that can be modified to address both the art (evocative) and science (didactic) of teaching in higher education. Technology offers opportunity for teachers to go outside the lifeworld of everyday teaching and learning and into creative, virtual and mixed reality worlds. Yet, it requires educators to maintain and develop their critical skills to become educational activists and essential agents of change. To help create and design the future of teaching and learning, educators can draw from their theoretical and practical experiences to evaluate their need to use digital technologies. In this way, they will be able to design learning activities that  prepare students to become creative and critical future-makers. 

## ’Who Owns the Future?’ Imagining Imagining from The Global South (Paul Prinsloo)

It is always good to start with a confession rather than a declaration and manifestos. The later will/may come later. To confess, whether under duress or by your own free will (if there is such a ‘will’), depends on the audience, considering the repercussions, considering what to share (and at what cost/benefit), and what to (rather) leave out (at what cost/benefit). This sounds like a confession about making a confession. But anyway, I digress.

I stole the first part of the title from a book by Jaron Lanier (2013). While it is a good, if not profound question, before we can consider/speculate about *owning* the future, it may be good to (rather) consider, *who* is allowed to, or claims the right to imagine or speculate about the future?

It reminds us of the words of Jean-Paul Sartre ‘Who dares to speak thus?’ (in the Preface to Fanon 2001: 8). The question asking who is laying claim to imagine the future, is, rather ironically, the inverse of the question, who has a right to define the *past*, the archive? (Derrida 1996 in Jacobsen and Beer 2021: 25) mooted that ‘there is no political power without control of the archive, if not of memory’. We therefore must consider also that there may be no political power without controlling what is imagined about the future. The archons not only record, maintain, and guard the archive, but also claim the hermeneutic right, competence, and ‘the power to interpret the archives’ (Derrida 1996 in Jacobsen and Beer 2021: 26). Likewise, those who claim the right to imagine the future, become not only the guardians of a particular imaginary but also distributors of a particular sensibility (Rancière 2010) of how the future may and should be understood. The right to define and imagine a particular world order is highly contested and as such, the ‘partition of the sensible’ is foundational in determining and enforcing who may participate and on what basis. Partitioning the sensible makes visible and hides, makes audible, and silences defines access and exclude, and is therefore ’a highly political act’ (Jacobsen and Beer 2021).

The sensibility-as-future-to-be resembles the notion of the social imaginary (Castoriadis 1987; Taylor 2002) as ‘organising mechanism’ to create public support and allocation of resources to *realise* such sensibility or imaginary. As such, imaginaries or ’organising visions’, ’technovisions’, ’technotales’, and a ’technomythscape’ (Dourish and Bell 2011: 1-2) resemble stories that are told about the future, ‘how the future will be achieved and realised …[but] exclude alternative visions and narratives’ about the future’ (Prinsloo 2020: 373). It is crucial to note that the partioning of the sensible also functions as generative matrices (Gaonkar 2002), ‘organising visions’, ‘technovisions’, ‘technotales’, and a ‘technomythscape’ (Dourish and Bell 2011: 1-2). Employed by national governments, these imaginaries function as ‘statecraft’ (Bebbington and McCourt 2007; Bulpitt 1986), and as there is evidence of how the techno-imaginations of supra-national organisations and alliances (e.g., UNESCO, the World Bank, the World Economic Forum), increasingly inform, and increasingly dictate national policy (McGinn 1994). We can therefore claim that these imaginaries are much more than ‘statecraft’ but actually ‘*futurecraft*’.

So, let us talk about imagining (technological futures). What would be the imaginings of ‘the wretched of the earth’ with their backs against the wall ready to ‘let loose at last that new violence which is raised up in [them] by old, oft-repeated crimes’ (Sartre in Fanon 2001: 26)? What are the imaginings of the ‘collateral casualties of progress’ (Bauman 2004: 29), the millions of the permanently homeless, refugees, and migrants, the outcasts of modernity? ‘The scholarly histories of technology and the digital are almost all intertwined with Western history, its theories, systems of knowledge production and its subsequent transfer’ (Bristow 2017: 281), which makes it very difficult to consider and/or imagine alternative, for example, African imaginings regarding a (technological) future.

Imagining technological futures from a positionality shaped by the African continent is difficult for two reasons. Firstly, as Bristow (2017) indicates, all imaginings about a technological future have already been colonized and the cost of being connected to technological futures imagined by others are paid in subscribing to the narratives of surveillance capitalism and data colonialization (Couldry and Mejias 2019). Secondly, it is very difficult to imagine alternative technological futures from African perspectives due to the reality that ‘scholarship on Africa did not adequately contain the subject of technology in Africa, and it was even rarer to find content in this field written from an African perspective’ (Bristow 2017: 283).

Evidence suggests that imaginations about Africa, and the Global South, in the Fourth Industrial Revolution are shaped by neoliberal and venture capitalists that dream and call forth particular African futures. There is therefore a need to decolonise these so-called futures and Bristow (2017) proposes talking about *post* African futures accounting for how current technological imaginations deepen structural inequalities while there is a need for imaginations of *repair*.

Can we therefore dare to imagine differently? Can we be courageous enough to lay claim to a particular sensibility (Rancière 2010) that focuses on *repair*, on a structural re-arrangement allowing the ‘wretched of the earth’ and the ‘collateral casualties of progress’ to be human, and become human? In conclusion, another confession. There is no declaration, no manifesto. There is only imagining differently.

## Conclusion (Juha Suoranta, Marko Teräs, Hanna Teräs)

We started this commentary by describing the recursive and all-encompassing narrative stating that ‘digitalization is shaping roles and practices in higher education’. With EdTech disruption speech, the main narrative of the current digitalization of higher education appears to describe the future and what is to be done to materialize it, usually with the help of technological development. In reality, the disruption speech manages to define only a future, and with enough repetition, it may end up being the hegemonic viewpoint, excluding different voices and constructing the future. Better yet, even when multiple voices are ‘engaged in development,’ they are usually forced to fit in the predetermined discourse, making it difficult to imagine other types of futures.

However, to avoid the apparent trap of predetermined and/or already colonized discourse, we need possibilities to imagine alternative and sustainable futures that ‘foster a critical imagination that is truly global and cosmopolitan in reach, and lives with doubt in the service of understanding’ (Back 2016: 131). In other words, we must study the options that change the normative parameters of current neoliberal discourse and the myth of technological solutions. Thus, the urgent task of speculative social science fiction methodology is to locate digitalization as part of the historical specificity of today’s HE policy trends. It is also to provide HE teachers and practitioners possibilities to imagine emancipatory and queer futures and create practical visions of hope. Besides, in the spirit of critical pedagogy, answer fundamental questions on the role of higher education in the face of planetary crises.

## Footnotes

1 See https://www.tandfonline.com/toc/cjem20/45/1. Accessed 27 September 2021.

2 The research project Speculative social science fiction of digitalization in higher education: Towards a theory and practice of humanized digital future (2021–2025) is funded by the Academy of Finland (343226). The project explores higher education teachers’ perspectives and trajectories and fosters awareness of their reflective pedagogical experiences and practical creativity in their future digitalized practices.

3 See https://www.oecd.org/education/2030-project. Accessed 27 September 2021.
